# Electrophysiological evidence of atypical visual change detection in adults with autism

**DOI:** 10.3389/fnhum.2013.00062

**Published:** 2013-03-06

**Authors:** H. Cléry, S. Roux, E. Houy-Durand, F. Bonnet-Brilhault, N. Bruneau, M. Gomot

**Affiliations:** UMR 930 Imagerie et Cerveau, Inserm, Université François Rabelais de ToursCHRU de Tours, France

**Keywords:** visual change detection, ERPs, vMMN, autism, adults

## Abstract

Although atypical change detection processes have been highlighted in the auditory modality in autism spectrum disorder (ASD), little is known about these processes in the visual modality. The aim of the present study was therefore to investigate visual change detection in adults with ASD, taking into account the salience of change, in order to determine whether this ability is affected in this disorder. Thirteen adults with ASD and 13 controls were presented with a passive visual three stimuli oddball paradigm. The findings revealed atypical visual change processing in ASD. Whereas controls displayed a vMMN in response to deviant and a novelty P3 in response to novel stimuli, patients with ASD displayed a novelty P3 in response to both deviant and novel stimuli. These results thus suggested atypical orientation of attention toward unattended minor changes in ASD that might contribute to the intolerance of change.

## Introduction

Increased attention has been paid in the past 10 years to the study of the event related potential (ERP) evoked by automatic change detection in the visual modality: the visual mismatch negativity (vMMN). This electrophysiological component has been extensively described in healthy adults as a negative component culminating over occipital sites between 150 and 350 ms in response to various deviant stimuli such as direction of movement (Kremlacek et al., 2006), form (Besle et al., 2005), orientation (Astikainen et al., 2008), spatial frequency (Maekawa et al., 2005), and color (Czigler et al., 2004). vMMN is thought to reflect the automatic pre-attentional detection of a difference between the active sensory memory trace of a recent repeated event (standard) and an incoming deviant stimulus (for review see Kimura, 2012), thus reflecting, as proposed in the auditory modality (Näätänen, 1995; Garrido et al., 2009), an online updating of the model for predicting sensory inputs. This response to automatic visual change is also known to be dependent on the degree-of-deviance as shorter MMN latencies have been recorded for greater deviant–standard differences (Czigler et al., 2002). Moreover, if the salience of change exceeds a certain threshold, MMN can be followed by an additional P3a component reflecting involuntary orientation of attention toward the rare event (Czigler, 2007).

vMMN has been investigated in several psychiatric disorders such as major depression (Chang et al., 2011; Qiu et al., 2011), schizophrenia (Urban et al., 2008), and cognitive decline (Tales et al., 2002a,b) which are characterized by sensory and cognitive dysfunction in several aspects such as attention memory and executive functions.

It is highly relevant to focus on automatic change detection in autism spectrum disorders (ASD) in the light of clinical evidence in individuals with ASD that they react in an unusual way to unattended events that occur in their environment or that prevent their routines. These atypical reactions may be expressed in the form of tantrums as a response to change, or in the form of restricted interests and repetitive or stereotyped behaviors, that persist with age (Kobayashi and Murata, 1998; Richler et al., 2010). Individuals with ASD try to impose predictability, with insistence on repetition and sameness (McEvoy et al., 1993). Resistance to change may also occur at the sensory level; individuals with ASD clinically display unusual behaviors in response to changes in all sensory modalities stimuli (Boyd et al., 2010). Moreover, several behavioral studies and results from questionnaires have revealed unusual sensory responses such as hyper-reactivity or hypo-reactivity in all sensory modalities (Khalfa et al., 2004; Leekam et al., 2007; Reynolds and Lane, 2008; Ashwin et al., 2009; Ben-Sasson et al., 2009), both sometimes occurring in the same subject. Such paradoxical responses to sensory stimuli have led to a lack of consensus on the exact nature of the underlying sensory dysfunction, but have been hypothesized to contribute to stereotyped behaviors and quest for sameness (Gerrard and Rugg, 2009). Moreover, study of relationships between clinical and electrophysiological findings has demonstrated that atypical brain reactivity in response to sensory changes occurring in stimulus sequence is related to the degree of behavioral intolerance of change as assessed by the Behavioral Summarized Evaluation (BSE-R, Barthelemy et al., 1997) (Gomot et al., 2011). As a whole, these features indicate that intolerance of change in ASD may be rooted in basic abnormalities in the processing of sensory information, and especially in the automatic processing of changing stimuli (Gomot and Wicker, 2012).

A substantial body of electrophysiological findings provides evidence for atypical processing of auditory change in ASD subjects compared to typically developing controls but the results in terms of MMN amplitude and latency have been inconsistent (for review see O'Connor, 2012). However, only one study has investigated the brain processes involved in automatic change detection in ASD using scalp potentials (SPs) and scalp current densities (SCDs) mapping (Gomot et al., 2002). This study showed shorter MMN latency in ASD associated with abnormal functioning of a neural network, including the left frontal cortex. These findings strongly suggest particular processing of auditory stimulus change in children with autism that might be related to their behavioral need to preserve sameness.

A few studies have investigated visual change detection in ASD *per se* but the protocols used have mostly involved active target detection (Kemner et al., 1994; Sokhadze et al., 2009). The majority of results indicated smaller P3 amplitude in response to novel visual events in those with ASD than in controls (Courchesne et al., 1989; Ciesielski et al., 1990). In a three stimulus oddball paradigm, Sokhadze et al. (2009) showed that ASD subjects displayed a delayed P3a response to visual novel stimuli, suggesting that individuals with ASD require more time to process the information needed for the successful differentiation of target and novel stimuli. These findings indicating differences in amplitudes and longer latencies in the electrophysiological index of attention-dependent novelty processing suggest unusual processing of violation of sensory expectancy in ASD, possibly due to difficulties in building flexible predictions about an upcoming event.

Maekawa et al. (2011) used a visual oddball paradigm comprising standard, deviant, and target windmill patterns in ASD. The participants were instructed to press a button when they recognized the target while they listened to a story delivered binaurally through earphones. The results revealed intact vMMN in terms of latency and amplitude in response to non-target deviants but a smaller P3 in response to targets. However, it can be argued that the mismatch response recorded in this study did not purely reflect pre-attentional processing of change detection, as stimuli were presented in the attentional visual field.

Only one study has investigated visual change detection in passive conditions in ASD (Cléry et al., 2013), using an oddball paradigm constituted of standard, deviant, and novel stimuli in children with ASD. Findings suggested that neural networks involved in the perception of visual changes in children with ASD are atypical and less modulated by the salience of stimuli than in typically developing children.

Thus no study to date has reported vMMN in adults with ASD in passive conditions. The aim of the study presented here was therefore to investigate automatic deviancy detection in the visual modality in adults with ASD in order to determine whether this pre-attentional ability was affected in this disorder. To verify whether the unusual sensibility of the neural networks involved in the perception of an even minor change is observable in adults with ASD, the same three stimuli oddball paradigm than in our previous study conducted in children (Cléry et al., 2013) was used. SPs and SCDs mapping was used to conduct spatio-temporal analyses of brain activation elicited by unattended changing visual stimuli.

## Materials and methods

### Participants

Thirteen adults with ASD (11 males and 2 females), aged 18 to 30 [mean age (years; months ± SD): 26; 2 ± 5] were recruited from the Autism Centre of the University Hospital of Tours. Diagnosis was made according to DSM-IV-R criteria (APA, 2000) and using the Autism Diagnostic Observation Schedule-Generic (ADOS-G, fourth module) (social interaction + communication scores mean ± SD: 10 ± 4; threshold for *ASD* = 7). Intelligence quotients (IQ) were assessed by the Wechsler Adult Intelligence Scale (WAIS-III). These intelligence scale provided overall intellectual (*mean* ± SD) (IQ: 89 ± 19), verbal (vIQ: 91 ± 17) and performance (nvIQ: 88 ± 24) quotients. Thirteen healthy volunteer also participated in the study [mean age (years; *months* ± SD): 24; 3 ± 2; 8 males and 5 females]. None of these healthy adults had a previous history of neurological or psychiatric problems. All participants had normal or corrected-to-normal vision and none were receiving psychotropic medication. The Ethics Committee of the University Hospital of Tours approved the protocol. Written informed consent from all participants was obtained.

### Stimuli and procedure

**Figure 1:**
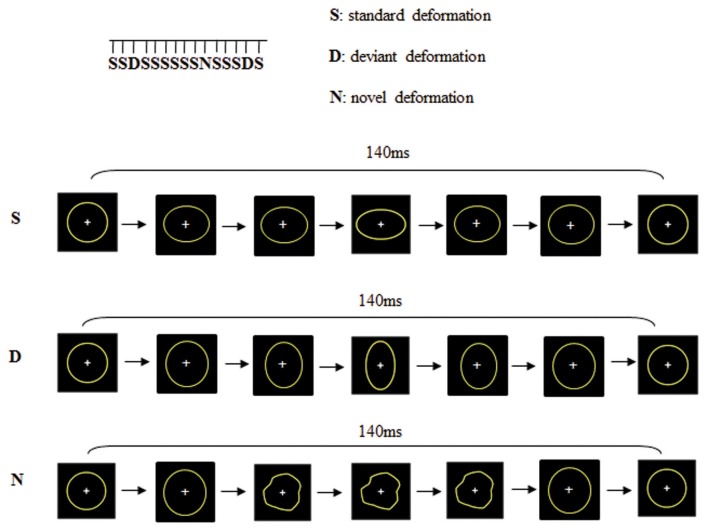
**Dynamic stimuli consisted on the deformation of a circle into an ellipse either horizontally (standard deformation) or vertically (deviant deformation) or into a new shape (novel deformation)**.

Change detection processes were studied using a passive visual oddball paradigm with three types of dynamic stimuli: “Standard” (probability of occurrence *p* = 0.82), “Deviant” (probability of occurrence *p* = 0.09) and “Novel” (probability of occurrence *p* = 0.09). As shown in Figure 1, these stimuli consisted in the deformation of a circle into an ellipse either horizontally (Standard) or vertically (Deviant) or into another shape (Novel), adapted from Besle et al. (2005). Each stimulus was constituted of seven successive images presented within 140 ms (i.e., 50 images per second) which resulted in apparent motions in the stimuli. The distinction between “deviants” and “novels” was not based on their probability of occurrence but on their salience. Whereas the deviant was always the same stimulus and only differed from the standard on the orientation of the ellipse, novel stimuli were always different non-identifiable shapes. Stimuli were presented with a 650 ms inter-stimulus interval. The viewing distance was set at 120 cm (visual angle 2°). There were 2 runs of 815 dynamic stimuli. To avoid confounds caused by physical features, Deviants were swapped with Standards halfway through the sequence. Total recording lasted 25 min. In order to present the visual stimuli within the visual field but outside the focus of attention, subjects were required to undertake a distractive task. They were asked to fixate the central cross (that appeared on the center of circles) and to respond as quickly as possible to its disappearance (Target 9% of the trials). The disappearance of the fixation cross (target) was never in synchrony with the presentation of deviant or novel stimuli but always during a standard trial.

### Acquisition and data analysis

The behavioral responses measured were mean reaction times (in ms) and response accuracy, calculated by taking into account the rates of hits (correct response less than 2 s after target disappearance), false alarms to non-target stimuli (response without target disappearance) and missed targets (no response within 2 s after target disappearance), according to the formula: (targets − missed targets)/(targets + false alarms) × 100 (Simon and Boring, 1990). Electroencephalographic (EEG) data were recorded from 31 Ag/AgCl electrodes referenced to the nose. Electrodes were placed according to the international 10–10 system (Chatrian et al., 1985): Fz, Cz, Pz, Iz, F3, C3, P3, O1, T3, T5, FC1, CP1, FT3, TP3, PO3 and their homologous locations on the right hemiscalp. Additional electrodes were placed at M1 and M2 (left and right mastoid sites), IM1 and IM2 (midway between M1-IZ and M2-IZ), and FFz (midway between Fz and Fpz). The whole experiment was controlled by a Compumedics NeuroScan EEG system (Synamps amplifier, Scan 4.3, and Stim2 software). The impedance value of each electrode was less than 10 kΩ. In addition vertical eye movements (EOG) were recorded using two electrodes placed above and below the right eye. The EEG and vertical EOG were filtered with an analog bandpass filter (0.3–70 Hz) and digitized at a sampling rate of 500 Hz. Eye-movement artifacts were eliminated using a spatial filter transform developed by NeuroScan. The spatial filter is a multi-step procedure that generates an average eye blink, utilizes a spatial singular value decomposition based on principal component analysis (PCA) to extract the first component and covariance values, and then uses those covariance values to develop a filter that retains the EEG activity of interest. EEG periods with movement artifacts were manually rejected. EEG epochs were averaged separately for the standard, the deviant and the novel stimuli over a 700 ms analysis period, including a 100 ms pre-stimulus baseline. The ERPs to deviants and novels included at least 120 trials for each subject. MMN was measured from the difference waves obtained by subtracting the standard-stimulus ERP from the deviant-stimulus ERP. Finally, responses to novelty were also examined by subtracting the standard-stimulus ERP from the novel-stimulus ERP.

The ELAN software package for analysis and visualization of EEG-ERPs was used (Aguera et al., 2011). Maximum amplitudes and peak latencies of the sensory ERP and mismatch responses were measured manually for each subject within a 80 ms time window around the peak of the grand average waveforms specific to each group.

SP maps were generated using a two-dimensional spherical spline interpolation and a radial projection from Oz (back views) or from Cz (top views), which respects the length of the meridian arcs. SCDs were estimated by computing the second spatial derivative of the interpolated potential distributions (Perrin et al., 1989). Topographic differences were specifically tested in the interactions between groups and electrodes on amplitude-normalized data (McCarthy and Wood, 1985). For each condition, measurements for each subject were normalized by finding the maximum and minimum values across all sites and by subtracting the minimum from each data point, and dividing it by the difference between maximum and minimum.

For each condition, amplitude and latency values were submitted to a mixed-model ANOVA with group (Controls vs. ASD) as the between subjects factor and electrode location [left vs. right Occipito-Parieto-Temporal regions (left OPT: O1, PO3, P3, T5; right OPT: O2, PO4, P4, T6)] as the within subjects factor. Within each group, the statistical significance of ERP amplitude compared to 0 was tested by student *t*-test analysis corrected for multiple comparisons, using the statistical-graphical method of Guthrie and Buchwald (Guthrie and Buchwald, 1991) as previously used in several electrophysiological studies (Colin et al., 2002; Vidal et al., 2008; Graux et al., 2012). This method provides a table indicating the minimum number of consecutive time samples that should be significant differences in ERP in order to declare an effect as significant over a given time period. For our sample of 13 subjects per group and an analysis period of 600 ms (from 0 to 600 ms, i.e., 300 sampling points), the minimum number corresponded to 12 consecutive time points (i.e., 24 ms) with *p*-values below the 0.05 significance level.

## Results

### Behavioral results

Both groups performed the distractive task well, indicating that all subjects have looked at the screen and thus received visual stimuli. Indeed, no significant between groups difference was found, neither in response accuracy (Ctrl: 95.2% ± 3.6; ASD: 94.4% ± 3.3; n.s.) nor in reaction times (Ctrl: 443 ms ±108; ASD: 475 ms ±77; n.s.).

### Electrophysiological analysis

**Figure 2:**
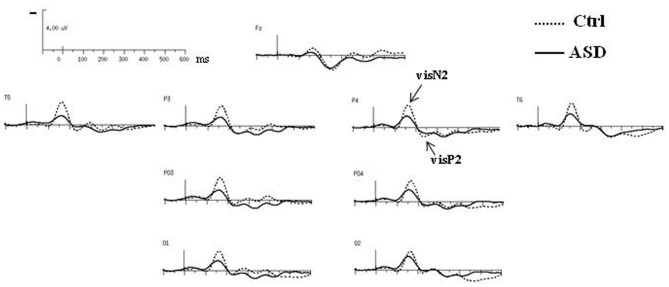
**Grand-average ERPs to the standard visual stimuli in both groups at selected electrodes**.

Both groups presented the same morphology and distribution of responses to standard visual stimuli, clearly localized over occipito-parietal sites, at O1, PO3, P3, T5 in the left hemisphere (left OPT) and at O2, PO4, P4, T6 in the right hemisphere (right OPT) (Figure 2). Unless specified, evaluations of left and right OPT responses were therefore calculated by averaging values measured at these four electrode sites on each hemisphere and statistical analyses of variance were conducted on these two sets of electrodes (left and right OPT as within subjects factor).

### Responses to standard stimuli

The obligatory responses consisted of a negative–positive complex peaking over parieto-occipital regions. In controls, a negative component peaked at a latency of 170 ms (called N2) and was followed by a more central positive wave culminating around 240 ms (P2) (Table 1). Compared to those of the controls, the responses in the ASD group to standard stimuli did not differ significantly in latency but displayed significant smaller amplitudes [N2: *F*_(2, 23)_ = 4.08, *p* < 0.05; P2: *F*_(2, 23)_ = 4.15, *p* < 0.05].

**Table 1:** **Mean amplitudes and latencies of the responses to standard, deviant, and novel visual stimuli in each group**.

			**Latence (ms ± SD)**			**Amplitude (μV ± SD)**		
			**Controls**		**ASD**	**Controls**		**ASD**
Standard	N2	L OPT	165 ± 18		161 ± 31	−2.5 ± 2.0	^*^	−1.1 ± 1.5
		R OPT	166 ± 19		156 ± 25	−2.5 ± 2.1	^*^	−1.5 ± 1.8
	P2	L OPT	236 ± 21		239 ± 37	1.7 ± 1.4	^*^	1.1 ± 1.0
		R OPT	236 ± 23		253 ± 29	1.9 ± 1.4	^*^	0.9 ± 0.6
Deviant	N2	L OPT	170 ± 16		161 ± 28	−2.5 ± 1.9	^*^	−1.2 ± 1.2
		R OPT	171 ± 17		156 ± 24	−2.5 ± 2.1	^*^	−1.5 ± 1.1
	P2	L OPT	269 ± 22	^*^	310 ± 33	1.4 ± 1.0		1.3 ± 1.2
		R OPT	274 ± 19	^*^	310 ± 34	1.5 ± 0.6		1.2 ± 1.3
Novel	Early N2	L OPT	194 ± 22		156 ± 27	−3.7 ± 3.1		−1.2 ± 1.2
		R OPT	190 ± 26		156 ± 22	−3.8 ± 2.8		−1.7 ± 1.2
	Late N2	L OPT	301 ± 27		–	−1.8 ± 2.3		–
		R OPT	304 ± 27		–	−1.8 ± 2.7		–
	P3	L OPT	434 ± 30		435 ± 32	2.9 ± 1.7		2.4 ± 2.0
		R OPT	431 ± 28		451 ± 36	2.6 ± 1.7		2.2 ± 1.8

* Significant between group difference p < 0.05.

### Responses to deviant and novel stimuli

**Figure 3:**
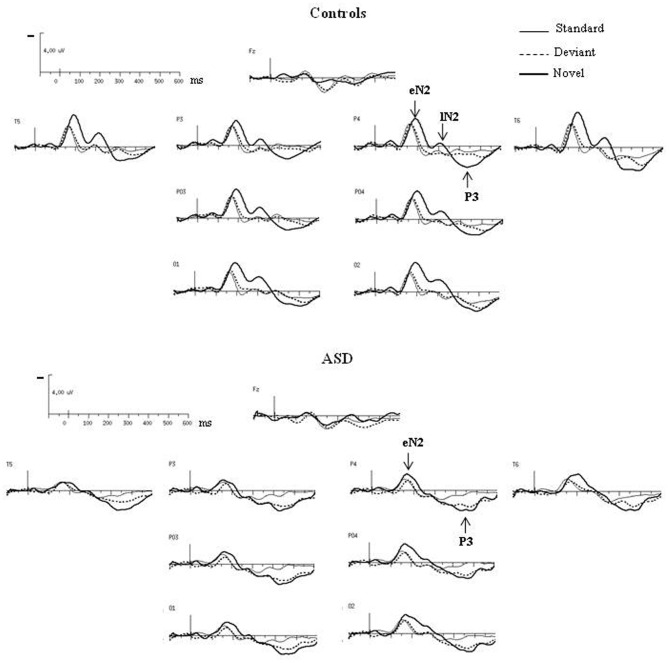
**Grand-average ERPs to the deviant and novel visual stimuli superimposed on the grand-average ERPs to the standard visual stimuli in both groups at selected electrodes**.

As shown in Figure 3, both groups had almost the same morphology and distribution of responses to the deviant as to the standard stimuli composed of a N2 peaking over occipito-parietal sites at left OPT and right OPT and a central P2. Compared to controls, ASD displayed significant smaller amplitudes of responses to deviant stimuli, but only for the N2 [*F*_(2, 23)_ = 3.57, *p* < 0.05]. Besides, the P2 in response to deviant is delayed in ASD [*F*_(2, 23)_ = 5.07, *p* < 0.05].

In response to novel stimuli, participants of the control group displayed a biphasic N2, peaking over occipito-parietal sites at left OPT and right OPT, first at 160 ms (early N2) and then at 320 ms (late N2), followed by a novelty P3 culminating at 440 ms (cf Table 1). Compared to controls, adults with ASD did not display comparable responses to visual novelty in term of morphology. Indeed, they only showed an early N2, also peaking over occipito-parietal sites at left OPT and right OPT at 160 ms. Both groups display similar early N2 topography as indicated by results of the mixed-model ANOVA: Group (Control vs. ASD) × Hemisphere (left, right) × Electrode site (Occipital, Parieto-Occipital, Parietal, Temporal) [*F*_(3, 72)_ = 0.27, n.s.]. This component was followed by a novelty P3 culminating at 440 ms. Neither the early N2 nor the novelty P3 showed significant between groups differences in terms of amplitude or latency (Table 1).

### Deviance processing

**Figure 4:**
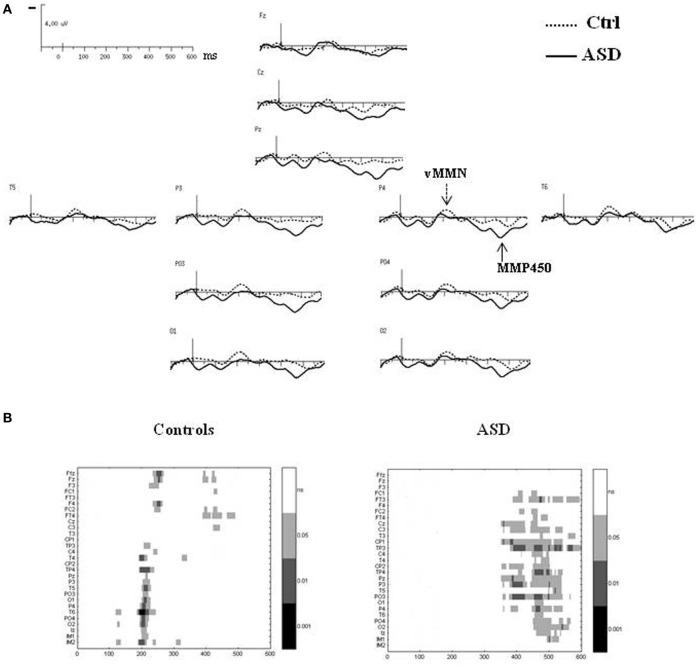
**(A)** Grand-average difference waves obtained by subtracting the ERPs to the standard stimuli from those to the deviant stimuli in each group at selected electrodes. **(B)** Paired student *t*-test analysis revealing statistical significance of the amplitude of the difference wave at 29 electrodes sites in the 0–600 ms latency range in controls (left panel) and in ASDs (right panel).

The difference waves were obtained by subtracting the standard-stimulus ERP from the deviant-stimulus ERP (Figure 4A).

In the control group, vMMN was elicited by the deviant stimuli, peaking over occipito-parietal sites at 210 ms (lOPT: 214 ms ± 22, −1.5 μV ± 1.0; rOPT: 210 ms ± 21, −1.6 μV ± 0.9; frontal: 226 ms ± 28, −1.1 μV ± 0.7) with a frontal negative deflection peaking later at around 230 ms. Figure 4B (left panel) shows the statistically significant amplitudes from 0 at 29 electrode sites between 0 and 600 ms post-stimulus in the adult group. Using the criteria defined in the “Materials and Methods” section, two periods of significant amplitude were distinguished: (1) from 180 to 240 ms after stimulus onset over occipito-parietal sites and (2) from 210 to 250 ms over fronto-central sites.

In adults with ASD (Figure 4A), a vMMN-like response was observed over occipito-parietal sites from 150 ms, followed as in controls by a frontal negative deflection peaking around 215 ms. Finally the automatic deviance detection process was completed by an additional significant positive component over occipito-temporo-parietal sites at 460 ms that we labeled Mismatch Positivity (MMP450) (lOPT: 1.55 ± 1.22 μV; rOPT: 1.58 ± 1.35 μV). However, results of the statistical analysis displayed in Figure 4B (right panel) indicated that in ASD only the MMP450 was statistically different from 0.

As both groups did not display similar significant components, direct group statistical comparison was not performed.

### Topographical analyses

#### Deviant–standard ERPs

**Figure 5:**
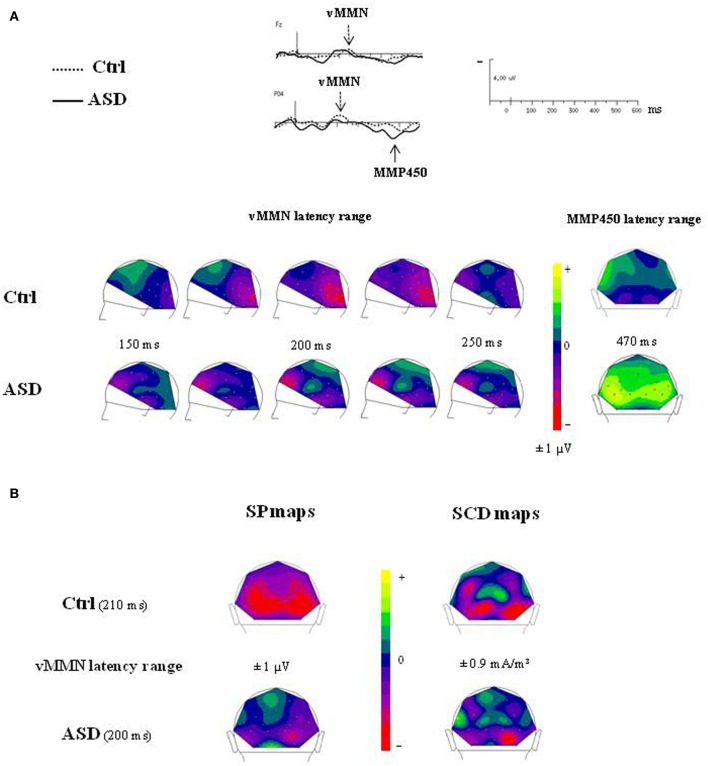
**(A)** Time course of the visual change detection process in the 150–250 ms latency range (left views) and SP maps of the peak latency of the MMP450 (back views) in both groups. **(B)** SP and SCD maps calculated in the vMMN lantecy range in both groups (back views).

The time course of the visual change-detection process in the 150–250 ms latency range is presented in Figure 5A for each group. The voltage maps in controls displayed negative potential fields over the bilateral occipito-parieto-temporal sites from 200 ms which reached the frontal region at around 230 ms. In the ASD group, SP maps showed a completely different time course of the visual change detection. Although non-significant, a first negative potential field was revealed over frontal site as soon as 150 ms, associated to a negative activity over infero-temporo-occipital sites, and from 200 ms an additional stable central positive activity was observed. Finally, SP maps calculated at the MMP450 peak latency showed in adults with ASD a large bilateral positive activity over the occipito-parietal areas whereas in controls no significant activity was measured.

The SCDs distributions of the change detection response at the latency of the vMMN for each group are shown in Figure 5B (bottom). SCD maps showed the involvement of both occipito-parietal and infero-temporo-occipital regions in both groups, as attested by the bilateral pattern of sinks recorded over occipital and parietal sites.

#### Comparison of deviant–standard and novel–standard ERPs

**Figure 6:**
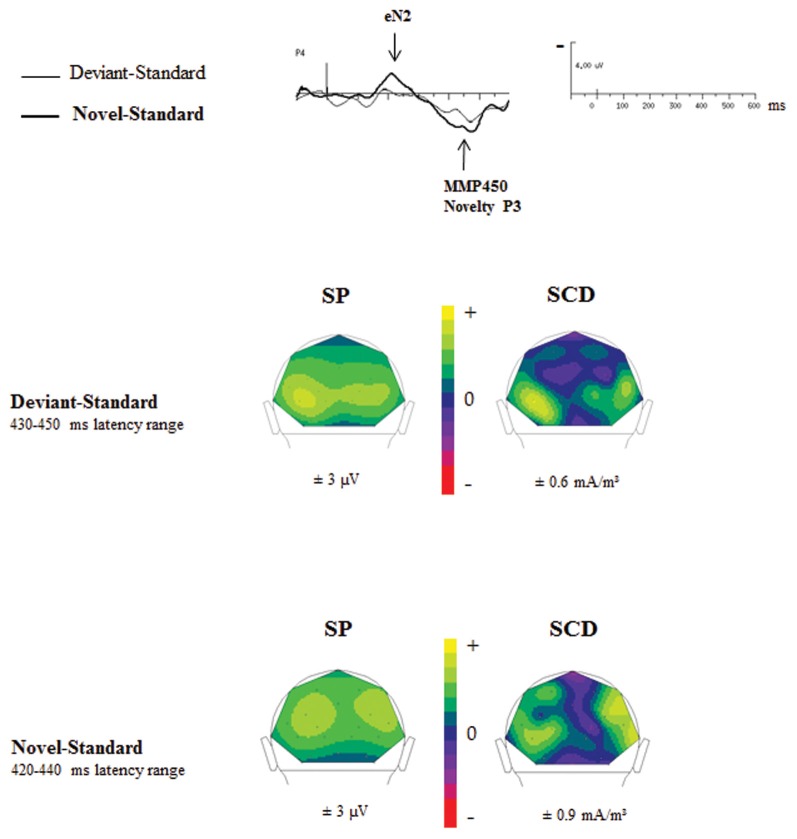
**Comparisons of the SP and SCD maps calculated in the MMP450 and the novelty P3 latency range in adults with autism**.

Figure 6 showed SP and SCD maps in ASD calculated in the latency range of the novelty P3 in response to novel (Novel–Standard ERPs) and of the MMP450 recorded in response to deviant stimuli (Deviant–Standard ERPs). SP maps showed for both responses a positive activity over bilateral occipito-parietal regions. SCD maps to both types of stimuli mainly showed bilateral occipito-parietal sources associated with a medial occipito-parietal current sink.

In order to determine whether the MMP450 (deviancy detection) and the novelty P3 (novelty detection) reflect the same component in ASD, we statistically compared the topographies of these two responses, using a mixed-model ANOVA: Condition (deviancy detection vs. novelty detection) × Hemisphere (left, right) × Electrode site (Occipital, Parieto-Occipital, Parietal, Temporal). ASDs display novelty P3 topography similar to that of the MMP450 as no significant topographic differences were found between these two conditions in this group [*F*_(3, 36)_ = 1.12, n.s.]. This indicates that MMP450 and novelty P3 represent the same response. Henceforth MMP450 in ASD should thus be labeled novelty P3.

## Discussion

The study presented here is the first to characterize electrophysiological indices of automatic visual deviancy processing in adults with ASD in passive conditions. Using a passive oddball paradigm, an atypical visual process was revealed in adults with ASD compared to controls.

The electrophysiological pattern of obligatory sensory responses to standard stimuli reported here showed the same morphology of response in both groups and consisted of a negative component peaking at around 170 ms (N2) followed by a positive component culminating at around 240 ms (P2). The N2 recorded here could reflect the main motion-onset visual evoked potential described by Kuba et al. (2007) peaking at around 150–200 ms and thought to be generated in the extrastriate temporo-occipital or parietal cortex (Nakamura and Ohtsuka, 1999; Henning et al., 2005). This N2 motion-onset is classically followed by a P2 deflection, usually peaking at around 240 ms and shown to depend on the type of motion presented (Kuba et al., 2007). These two sensory responses displayed significantly reduced amplitude in adults with ASD than in controls. Such smaller amplitudes were similarly observed in response to deviant visual stimuli. It should be noted that the visual stimuli used consisted of the dynamic deformation of a circle into an ellipse in either one or another direction, resulting in two different shapes and thus involving two visual dimensions: object shape and motion direction. This kind of visual stimuli involving changes in form and motion was chosen to increase the chances of obtaining vMMN by stimulating the mismatch process with two physical stimulus features. Indeed, the visual system is functionally divided into at least two pathways (for review see Farivar, 2009). The ventral pathway is generally specialized for fine detail, static form, and color perception, whereas the dorsal pathway is predominantly responsible for processing and perceiving moving stimuli, locating objects and directing visually guided action. A number of studies have reported low-level perception deficits in ASD, mainly characterized by higher motion coherence thresholds, but intact performance on form coherence tasks, suggesting a specific dysfunction of the visual dorsal pathway (Spencer et al., 2000; Milne et al., 2002; Braddick et al., 2003). The hypothesis of specific dorsal stream vulnerability in ASD has been questioned by findings suggesting an additional ventral stream deficit in ASD (Spencer and O'Brien, 2006) using a spatial-form-coherence detection task. The specific features of our dynamic stimuli could explain the atypical morphology of the sensory response in ASD, as numerous studies pointed to abnormalities in coherent motion perception and in local motion processing in ASD (for review see Simmons et al., 2009). Nevertheless, despite the large number of studies published on visual ERPs in autism, direct comparison of our results with previous findings is not easy as, to our knowledge, no study has reported ERPs in response to stimuli similar to those used in this study.

Visual MMN was identified in the control group, culminating over occipito-parietal sites at around 210 ms, followed by an anterior negative component peaking at 230 ms. This finding confirms previous studies suggesting the location of vMMN generators in both the visual occipital (Czigler et al., 2004; Pazo-Alvarez et al., 2004; Amenedo et al., 2007) and the frontal areas (Czigler et al., 2004; Urakawa et al., 2010). In adults with ASD, the visual MMN was almost absent. However, in view of the SP and SCD maps, it cannot be excluded that adults with ASD displayed a mismatch process comparable to that of the controls, but of smaller amplitude that did not reach significance. All the studies that have investigated vMMN in psychiatric disorders characterized by sensory and cognitive dysfunctions (for review see Maekawa et al., 2012) have revealed a significantly smaller vMMN in psychiatric patients than in controls. Taken together, these results suggest that an impaired vMMN generation might contribute to characterize elementary cognitive processing in psychiatric disorders.

In ASD, the mismatch response was mainly characterized by a significant positive component culminating over bilateral occipito-parietal sites at around 460 ms and that we first labeled MMP450. Increasing the salience of visual change by presenting novel stimuli evoked a biphasic negative deflection (early N2 and late N2) followed by a positive novelty P3 component in controls. Adults with ASD did not display the same morphology of responses to novel stimuli as they only showed an early N2 followed by a novelty P3. Interestingly, the MMP450 recorded in response to deviance and the novelty P3 recorded in response to novel stimuli in ASD appeared at similar latencies and displayed the same scalp topography, thus suggesting that they reflect the same process. Because novelty P3 is thought to reflect involuntary switching of attention toward stimulus changes occurring outside the focus of attention (Pontifex et al., 2009), it can be hypothesized that adults with ASD are more attracted than controls by any visual change (even non-significant) occurring unexpectedly in their environment. This finding of a large novelty P3 in response to deviant stimuli is in accordance with our study investigating automatic visual change detection in children with ASD using the same paradigm (Cléry et al., 2013) and supports clinical reports showing that individuals with ASD often tend to be more distractible than controls, suggesting that their attention may in fact be “underselective” (Allen and Courchesne, 2001; Keehn et al., 2012). This may explain why individuals with ASD appear to ignore relevant stimuli in the environment in favor of relatively discrete and apparently meaningless stimuli, but it may also contribute to the exceptional perceptual abilities observed in some individuals with ASD (Mottron et al., 2006; Plaisted-Grant and Davis, 2009). This might be a maladjustment in so far as it leads to distress at small changes in the environment (Happe and Frith, 2006).

Interestingly patients with ASD displayed a smaller (non-significant) vMMN than controls in response to deviant stimuli, leading to suggest poorer automatic visual change detection in this pathology, but followed by an additional large novelty P3 reflecting the involuntary switching of attention toward stimulus changes. This finding raised question about the possible dissociation of this two components as it remains surprising that the attention could be involuntary captured by a change, without this change being first detected. However, similar cases of dissociation between early change detection negativity and the subsequent P3 have been reported in the auditory modality (Winkler et al., 1998; Sussman et al., 2003; Rinne et al., 2006). Recently Horváth et al. (2008) investigated distraction-related ERP responses using an auditory distraction paradigm and showed that a P3a can be elicited without previous MMN in response to some stimulus features. The authors proposed that the P3a may rather reflect some possibly higher-level event detection process than attention switching itself. Such observation merits further investigations in the visual modality.

This finding that even small deviance detection involved a novelty P3 response in adults with ASD may be related to results previously obtained in children in the auditory modality by Gomot et al. (2002). Taken together these findings support of the existence of an atypical change detection process acting in several sensory modalities in people with ASD that might contribute to their intolerance of change.

### Conflict of interest statement

The authors declare that the research was conducted in the absence of any commercial or financial relationships that could be construed as a potential conflict of interest.
